# A patient with metastatic melanoma presenting with gastrointestinal perforation after dacarbazine infusion: a case report

**DOI:** 10.1186/1752-1947-4-10

**Published:** 2010-01-15

**Authors:** Sjoukje F Oosting, Frans TM Peters, Geke AP Hospers, Nanno H Mulder

**Affiliations:** 1 Department of Medical Oncology, University Medical Center Groningen and University of Groningen, 9700 RB, Groningen, The Netherlands; 2 Department of Gastroenterology, University Medical Center Groningen and University of Groningen, 9700 RB, Groningen, The Netherlands

## Abstract

**Introduction:**

We report a rare case of gastrointestinal perforation following dacarbazine infusion for metastatic melanoma. The condition is attributed to a responding malignant melanoma in the gastrointestinal tract.

**Case presentation:**

A 52-year-old Caucasian man presented with abdominal pain and distension, malaise, night sweats, dysphagia and early satiety. A computed tomography scan showed massive ascites, lymphadenopathy and liver lesions suspect for metastases. An upper gastrointestinal endoscopy was performed and revealed multiple dark lesions of 5 mm to 10 mm in his stomach and duodenum.

When his skin was re-examined, an irregular pigmented lesion over the left clavicle measuring 15 mm × 8 mm with partial depigmentation was found. Histological examination of a duodenal lesion was consistent with a diagnosis of metastatic melanoma. The patient deteriorated and his level of lactate dehydrogenase rapidly increased. The patient was started on systemic treatment with dacarbazine 800 mg/m^2 ^every three weeks and he was discharged one day after the first dose. On the sixth day he was readmitted with severe abdominal pain. A chest X-ray showed the presence of free intraperitoneal air that was consistent with gastrointestinal perforation. His lactate dehydrogenase level had fallen from 6969U/L to 1827U/L, supporting the conclusion that the response of gastrointestinal metastases to dacarbazine had resulted in the perforation of the patient's bowel wall. A laparotomy was discussed with the patient and his family but he decided to go home with symptomatic treatment. He died 11 days later.

**Conclusion:**

Melanoma can originate in, as well as metastasize to, the gastrointestinal tract. Gastrointestinal perforations due to responding tumors are a well-known complication of systemic treatment of gastrointestinal lymphomas. However, as the response rate of metastatic melanoma to dacarbazine is only 10% to 20%, and responses are usually only partial, perforation due to treatment response in metastatic melanoma is rare.

Medical oncologists should be aware of the risk of bowel perforation after starting cytotoxic chemotherapy on patients with gastrointestinal metastases.

## Introduction

The incidence of melanoma is increasing worldwide. In The Netherlands 19.4 cases per 100,000 persons were diagnosed in 2005. For the treatment of widespread metastatic diseases, single agent dacarbazine (DTIC) chemotherapy is still the standard of care. Combination regimens with other cytotoxic agents, cytokines and tyrosine kinase inhibitors do not result in a survival benefit [1-3].

Treatment with high-dose interleukin-2 (IL-2) has induced a durable complete remission in a minority of patients with metastatic melanoma, but this treatment is associated with severe toxicity and it is not widely available [4].

Treatment with dacarbazine results in response rates of 10% to 20%. Responses are usually partial and generally last for only four to six months, although prolonged remissions are occasionally seen. A survival benefit of treatment with dacarbazine over best supportive care has not been proven definitively [5]. Compared to other cytotoxic agents, dacarbazine is relatively well tolerated. Nausea is the most frequent side effect, however, this is easily controllable with modern anti-emetics.

## Case presentation

In November 2007, a 52-year-old Caucasian man of Dutch origin presented with upper abdominal pain, anorexia, nausea, dyspnea on exertion, and a general decline in condition for the past few weeks. His medical history revealed a subarachnoid hemorrhage eight years prior to presentation, from which he recovered completely, and essential hypertension that was well-controlled.

**Figure 1:**
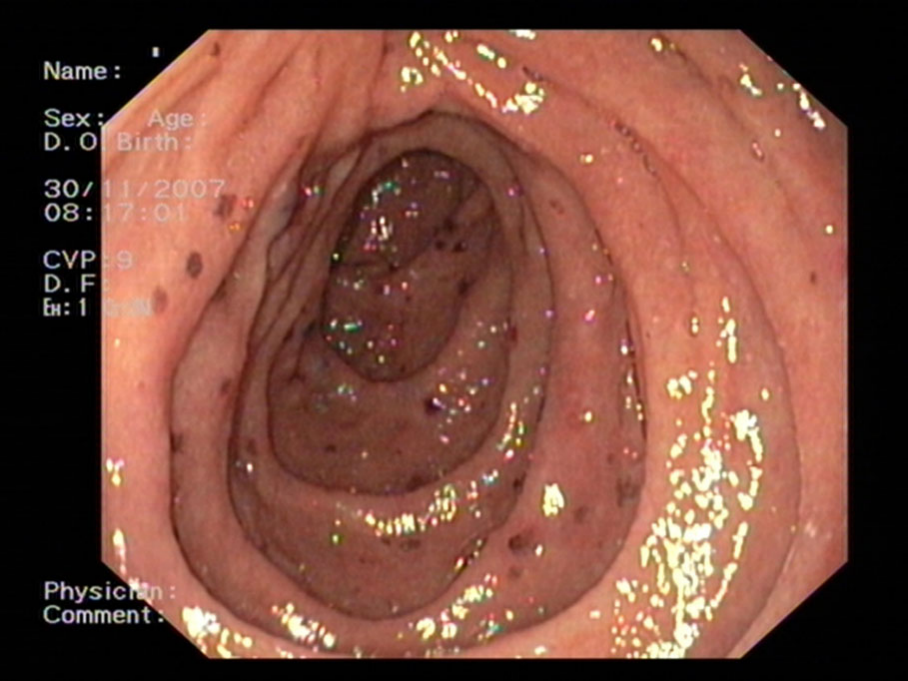
**Upper gastrointestinal endoscopy showing multiple dark duodenal lesions measuring 5 mm to 10 mm**.

**Figure 2:**
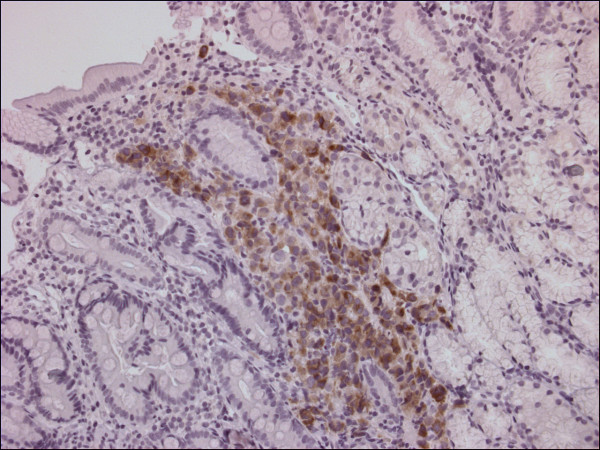
**Histological examination of a duodenal lesion, Melan A staining**.

On physical examination, a lymphadenopathy in the patient's left axilla and neck was found, in combination with a distended abdomen with shifting dullness and an enlarged irregular liver. Laboratory tests showed a slight leukocytosis and thrombocytosis, normal haemoglobin, creatinine and electrolytes levels, a lactate dehydrogenase (LDH) level of 373IU/L that increased to 6969IU/L in eight days, normal alkaline phosphate, normal transaminases and bilirubins. A computed tomography (CT) scan of his chest and abdomen revealed lymphadenopathy in the mediastinum, lung hili and left axilla, as well as ascites with an omental cake and multiple lesions in an enlarged liver. Ascitic fluid was sent to pathology, and a gastroduodenoscopy was also performed. Multiple dark gastric and duodenal lesions were found, which were suspect for metastatic melanoma or Kaposi's sarcoma (Figure 1). A biopsy of one of these lesions was consistent with melanoma (Figure 2), as was the cytological analysis of the ascitic fluid.

**Figure 3:**
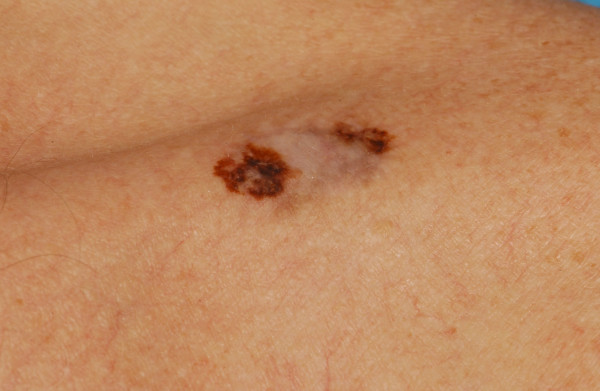
**Pigmented skin lesion over the left clavicle measuring 15 mm × 8 mm with partial depigmentation consistent with a melanoma in regression**.

Subsequently, on re-examination of the skin, a 1.5 cm irregular lesion over the left clavicle was found. The lesion was partially pigmented and partially depigmented, which was consistent with a melanoma in regression (Figure 3).

Two days after an uncomplicated paracentesis, our patient received 1560 mg (800 mg/m^2^) dacarbazine intravenously. He was discharged the next day.

**Figure 4:**
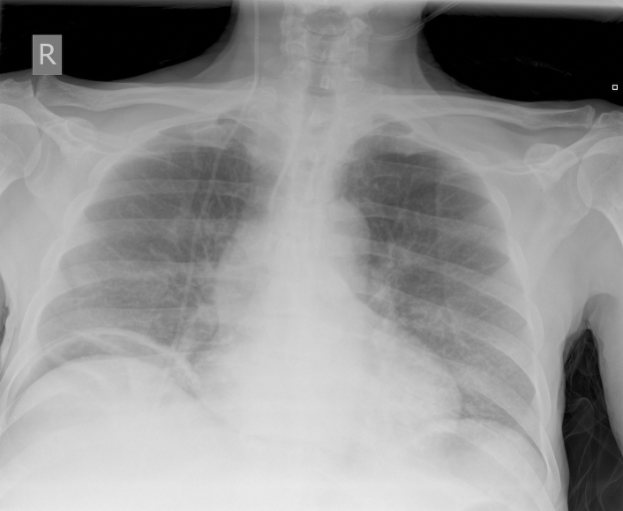
**Chest X-ray showing free intraperitoneal air**.

Five days after chemotherapy he was readmitted with severe abdominal pain. On physical examination, an acute abdomen was found. A chest X-ray showed the presence of free intraperitoneal air (Figure 4) and a clinical diagnosis of gastrointestinal perforation was made. His serum LDH level had fallen to 1827IU/L. A nasogastric tube was given and the patient was started on broad spectrum antibiotics. The benefits and risks of laparotomy were discussed with the patient and his family, and he decided to go home with supportive care. He died 11 days after the diagnosis of gastrointestinal perforation was made.

## Discussion

Melanoma can originate anywhere in the digestive tract, but the majority of digestive tract lesions are metastatic in nature. The primary lesion can be discrete, especially when, as in this case, regression has occurred. In patients with end stage melanoma, gastric metastases and small bowel metastases are quite common. In two large autopsy series, gastric metastases were found in about 20% of the patients studied, and small bowel metastases in 35.6% and 58% of the patients [6,7].

Differentiation between a primary gastrointestinal tract lesion and metastasis from an occult cutaneous melanoma can be difficult in cases with solitary gastrointestinal localization. However, due to the lack of convincingly effective adjuvant regimens for melanoma, the clinical consequences are marginal.

Surgery for melanoma lesions that are metastatic to the gastrointestinal tract is quite effective for controlling symptoms but it rarely leads to long-term survival [8,9].

The systemic treatment of metastatic disease has been met with limited success. Over the decades, single-agent treatment with dacarbazin has remained the standard of care. An incentive for using this drug even in the end stage of the disease is its limited toxicity, with easily controllable nausea as its main clinical side effect. The generally mild hematological toxicity of dacarbazine compares favorably to that of drugs used in other advanced stages of cancer [10]. Although the response rate is low, prolonged remissions are sometimes achieved, which makes treatment worthwhile.

Perforation of a gastrointestinal tumor as a result of chemotherapy is rare, as remissions are usually partial and occur gradually. An exception in this regard is intra-abdominal lymphoma. This tumor is highly sensitive to cytotoxic therapy and responds with rapid necrosis. Abdominal non-Hodgkin's lymphoma in children, with otherwise favorable prognosis, is associated with a very poor outcome if gastrointestinal perforation occurs [11,12].

We could only identify one other case of fatal gastrointestinal perforation in a patient with metastatic melanoma during treatment with dacarbazine, but bowel perforation was not attributed to chemotherapy by the authors [10]. In our patient, perforation was ascribed to responding gastrointestinal metastases. A recent gastrointestinal endoscopy had revealed multiple metastases and no peptic ulcers. Our patient had not been using non-steroidal anti-inflammatory drugs (NSAIDs), which makes perforation of a gastric or duodenal ulcer unlikely. Furthermore, his LDH level had fallen dramatically, thus indicating his fast response to chemotherapy.

## Conclusion

Chemotherapy in end stage melanoma is aimed at a rarely achievable goal of prolonged remission in the context of limited toxicity. In the presence of extensive digestive tract involvement, a remission could lead to perforation, resulting in excess toxicity and probably death. When perforation is recognized as a possible threat, a prolonged administration of the drug in its oral formulation might be advisable. This does not seem to compromise treatment efficacy [10].

## Consent

Written informed consent was obtained from the partner of the patient for publication of this case report and any accompanying images. A copy of the written consent is available for review by the Editor-in-Chief of this journal.

## Competing interests

The authors declare that they have no competing interests.

## Authors' contributions

SO treated the patient with chemotherapy and drafted the manuscript. FP performed the endoscopy and made the diagnosis of metastatic melanoma. GH conceived of the manuscript and participated in its design. NM supervised this patients treatment and drafted the manuscript. All authors read and approved the final manuscript.
